# Quantification of the ozone and singlet delta oxygen produced in gas and liquid phases by a non-thermal atmospheric plasma with relevance for medical treatment

**DOI:** 10.1038/s41598-018-30483-w

**Published:** 2018-08-15

**Authors:** Helena Jablonowski, Joao Santos Sousa, Klaus-Dieter Weltmann, Kristian Wende, Stephan Reuter

**Affiliations:** 1 ZIK plasmatis at Leibniz Institute for Plasma Science and Technology e.V. (INP Greifswald e.V.), Felix-Hausdorff-Str. 2, 17489 Greifswald, Germany; 2 LPGP, CNRS, Univ. Paris-Sud, Université Paris-Saclay, 91405 Orsay, France; 3 Leibniz Institute for Plasma Science and Technology e.V. (INP Greifswald e.V.), Felix-Hausdorff-Str. 2, 17489 Greifswald, Germany

## Abstract

In the field of plasma medicine, the identification of relevant reactive species in the liquid phase is highly important. To design the plasma generated species composition for a targeted therapeutic application, the point of origin of those species needs to be known. The dominant reactive oxygen species generated by the plasma used in this study are atomic oxygen, ozone, and singlet delta oxygen. The species density changes with the distance to the active plasma zone, and, hence, the oxidizing potential of this species cocktail can be tuned by altering the treatment distance. In both phases (gas and liquid), independent techniques have been used to determine the species concentration as a function of the distance. The surrounding gas composition and ambient conditions were controlled between pure nitrogen and air-like by using a curtain gas device. In the gas phase, in contrast to the ozone density, the singlet delta oxygen density showed to be more sensitive to the distance. Additionally, by changing the surrounding gas, admixing or not molecular oxygen, the dynamics of ozone and singlet delta oxygen behave differently. Through an analysis of the reactive species development for the varied experimental parameters, the importance of several reaction pathways for the proceeding reactions was evaluated and some were eventually excluded.

## Introduction

Cold physical plasma jets have shown a high potential in the treatment of chronic wounds^1–3^ and in cancer as an adjuvant to standard therapy^4^. Further targets are currently investigated, e.g. atopic eczema^5^. Common to all these applications is that reactive oxygen species (ROS) have relevant impact via redox signalling processes^6,7^. Besides electromagnetic radiation (ultraviolet - UV - up to near infrared - NIR - spectral range), atmospheric pressure plasma jets generate various reactive oxygen and reactive nitrogen species (RNS), such as ozone, singlet delta oxygen, hydroxyl radicals, superoxide anion radicals, nitric monoxide, nitrogen dioxide, nitrite, and nitrate^8^. Most of these species are thought to interact directly or indirectly with biological systems, e.g. cells in a human body. In order to tailor the plasma composition for a given medical application, it is essential to characterize the plasma, the reactive species deposited in liquids, and the subsequent biological effects of those reactive species. Hence, knowing the details of the transfer of the reactive species from the gas phase into liquids helps the interpretation of biological data and is useful to the construction of dedicated plasma sources. It also helps to evaluate the suitability of remote treatment approaches for medical usage that are being studied with increasing emphasis. Although increasing knowledge is available on time- and spatially resolved density distribution of reactive species in the gas phase, their trajectories in a liquid system remain elusive^8–11^. The formation pathway of many species in the liquids is not clear: liquid phase species can either be generated by a direct interaction of the plasma with the liquid at the interface^12^, e.g. due to (vacuum)UV-caused photo dissociation of water molecules^13,14^, or alternatively, via transfer from the gas phase into the liquid^15^, or even by secondary or tertiary reactions in the bulk liquid^16^. One of the few species whose formation pathway in the liquids is well studied is hydrogen peroxide in the case of plasma jets fed with humidified argon^15,17^ and helium^18^ gases. Shortcomings in specificity or sensitivity limit bar the quantification of most other species in liquids. Short lifetimes or rapid conversions further impede quantification^19^. Some more recent approaches start to overcome these limits^12,19–22^. Models predict a large role of the interface layer, with impact on species attachment and solvation into the bulk liquid^12,23–27^. Yet, knowledge on the species distribution in the bulk liquid, especially when in contact with a biological surface or in presence of organic molecules, is very limited^23,24,28^. On the other hand, a large body of evidence exists, showing the impact of plasma derived species in biological systems, proving the permeation of the aqueous barriers, like physiologic buffer systems, cell culture media, or body fluids^10,11,29–36^.

At times, specific plasma-generated species have been attributed to trigger the stimulation, modulation, or execution of the biological effects observed. Among these are hydrogen peroxide (H_2_O_2_)^15,17^, atomic oxygen (^•^O)^37^, or nitric oxide (^•^NO)^38^ and peroxynitrite (ONOO^−^)^16,22,39^. Other species are harder to pinpoint, although present in high amounts in the gas phase. This is valid for plasma generated ozone, O_3_^40^, and singlet delta oxygen, O_2_(a^1^Δ_g_)^41^, whose full biological impact in plasma medicine is far from being completely known. O_3_ is a powerful oxidant which is well known for disinfection of drinking water^42^. In this case, O_3_ is the precursor for even stronger oxidants such as the hydroxyl radical (^•^OH)^43^. The lifetime of O_3_ is relatively long; in water, O_3_ has a half-life of seconds up to hours^44^, depending on the temperature and the pH value^45^. Hence, in solution, the lifetime of O_3_ is much longer than the half-life of ^•^OH, which is of the order of ns^46^. In the gas phase, O_3_ is even more stable; its half-life is in the range of hours up to days^47^, depending on the temperature, humidity and air speed. O_2_(a^1^Δ_g_) is also a highly reactive ROS. In comparison to O_3_, it is less stable in both the gas and the liquid phases. Its half-life is 75 minutes in the gas phase^41^ and several µs in the liquid phase^48^. Nevertheless, O_2_(a^1^Δ_g_) is still more stable than most oxygen radicals. In the presence of superoxide anion radicals, O_2_^•−^, O_2_(a^1^Δ_g_) is quenched to molecular oxygen^49^. If H_2_O_2_ and hypochlorite (OCl^−^) are simultaneously present in the solution, O_2_(a^1^Δ_g_) can be generated directly in the liquid^50^. Biological effects of O_2_(a^1^Δ_g_) are evident in the context of the photodynamic therapy^51^.

In the gas phase, the variation of O_2_(a^1^Δ_g_) and O_3_ densities as a function of the O_2_ admixture to the feed gas has already been shown for several plasma sources^41,52–55^. Here, the O_2_(a^1^Δ_g_) as well as the O_3_ concentrations are determined, for the first time, in the liquid and in the gas phase under identical conditions. To study the origin and transfer between the gas and liquid phases of plasma generated O_3_ and O_2_(a^1^Δ_g_), a well-characterized argon plasma jet was used (kinpen09)^17,20,56,57^. By using a curtain gas device, the ambient conditions were controlled^58^, allowing the modulation of the gas composition around the plasma plume. The O_2_ content of the working gas was also regulated. This approach allows tuning the reactive species composition in the solution and increases the treatments reproducibility^17,59–61^, forming the base for the presented data. The final aim of the present paper is to estimate the potential biological impact of these two ROS, O_3_ and O_2_(a^1^Δ_g_), and to evaluate the suitability of direct and indirect treatment procedures.

## Results

### Reactive oxygen species in the liquid

During the plasma treatment of DPBS (Dulbecco’s phosphate buffered saline solution), several reactive oxygen species are induced in the liquid^10^: O_3_, ^•^O, or O_2_(a^1^Δ_g_).

**Figure 1:**
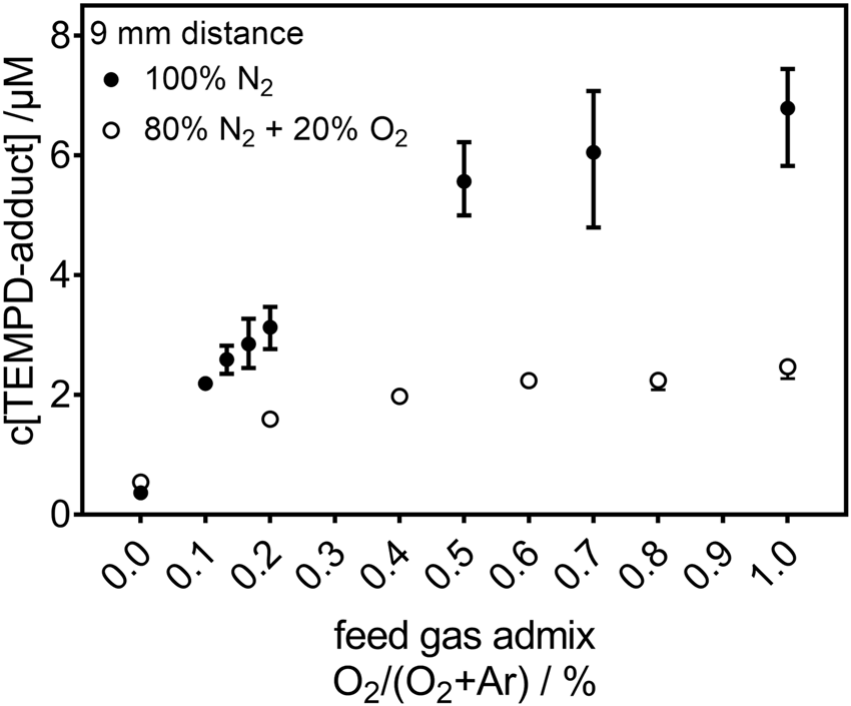
Concentration of spin probe TEMPD-HCl adducts after direct plasma treatment (9 mm, 180 s). In both environmental conditions (N_2_ or air-like), plasma induces ROS in the liquid. The TEMPD-HCl adduct of O_3_, ^•^O, O_2_(a^1^Δ_g_) is depicted as function of the O_2_ content in the feed gas of Ar.

In Fig. 1, the concentration of the spin probe TEMPD-HCl adducts is given as a function of the O_2_ content in the feed gas for two different curtain gas conditions (pure N_2_ and synthetic air). This spin probe adduct is formed by a reaction with either O_3_, ^•^O, or O_2_(a^1^Δ_g_). The composition of the gas surrounding the plasma plume influences the spin probe adduct concentration. Air-like conditions lead to a lower adduct-concentration than the pure N_2_ curtain gas. Furthermore, the presence of O_2_ in the surrounding yields a saturation of the adduct concentration for O_2_ contents in the argon higher than 0.4%. For a curtain gas of N_2_, there is always an increase of the adduct concentration, but at a lower rate for higher O_2_ content in the feed gas – no clear saturation is observed. As this spin probe results in the same EPR spectrum for the three potential reactants (O_3_, ^•^O, or O_2_(a^1^Δ_g_)), further measurements are necessary in order to distinguish the species and identify their origin.

**Figure 2:**
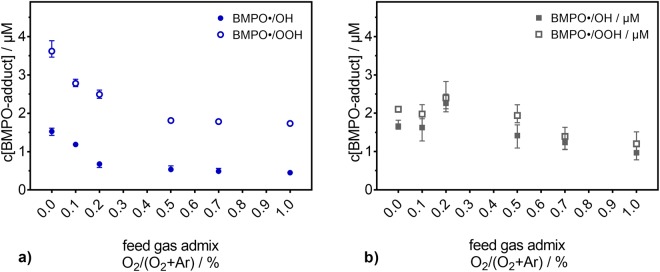
Concentration of free oxygen radical spin trap (BMPO) adducts after direct plasma treatment (9 mm, 180 s). The formation of the ^•^OH (filled symbols) and O_2_^•−^ (open symbols) adducts is investigated as a function of the O_2_ content in the feed gas of Ar under (**a**) pure N_2_ or (**b**) air-like atmospheres.

^•^OH recombines in a diffusion-controlled manner to H_2_O_2_. Moreover, O_2_^•−^ can also disproportionate to H_2_O_2_. Therefore, the H_2_O_2_ concentration was determined for the two curtain gas compositions. In Fig. 3a, the concentration of H_2_O_2_ in the liquid is given as a function of the O_2_ content in the feed gas. A similar trend to those of the concentrations of ^•^OH and O_2_^•−^ radicals was observed (if one do not consider the first two points for an air-like atmosphere).

The behaviour of ^•^OH and O_2_^•−^ for similar experimental conditions was also studied. Using BMPO, ^•^OH and O_2_^•−^ were detected for pure N_2_ as curtain gas (see Fig. 2a) and an air-like atmosphere (see Fig. 2b). The resulting spin trap adducts, BMPO-OH and BMPO-OOH respectively, show an entirely different behaviour compared to the TEMPD-HCl adduct. However, the concentrations of both BMPO adducts have the same trend. For pure N_2_ as curtain gas, their concentrations decrease with increasing O_2_ content in the feed gas, and the strongest impact on the formation of the BMPO adducts occurs for low amounts of O_2_: pure argon yields the highest concentration of BMPO adducts, which decreases drastically with the addition of 0.2% of O_2_ or more. For an air-like atmosphere, the variation of the concentration of BMPO adducts as a function of the O_2_ admixture into the feed gas is not so pronounced, with maxima concentrations being obtained for 0.2% of O_2_ and the lowest concentrations for the higher O_2_ content.

**Figure 3:**
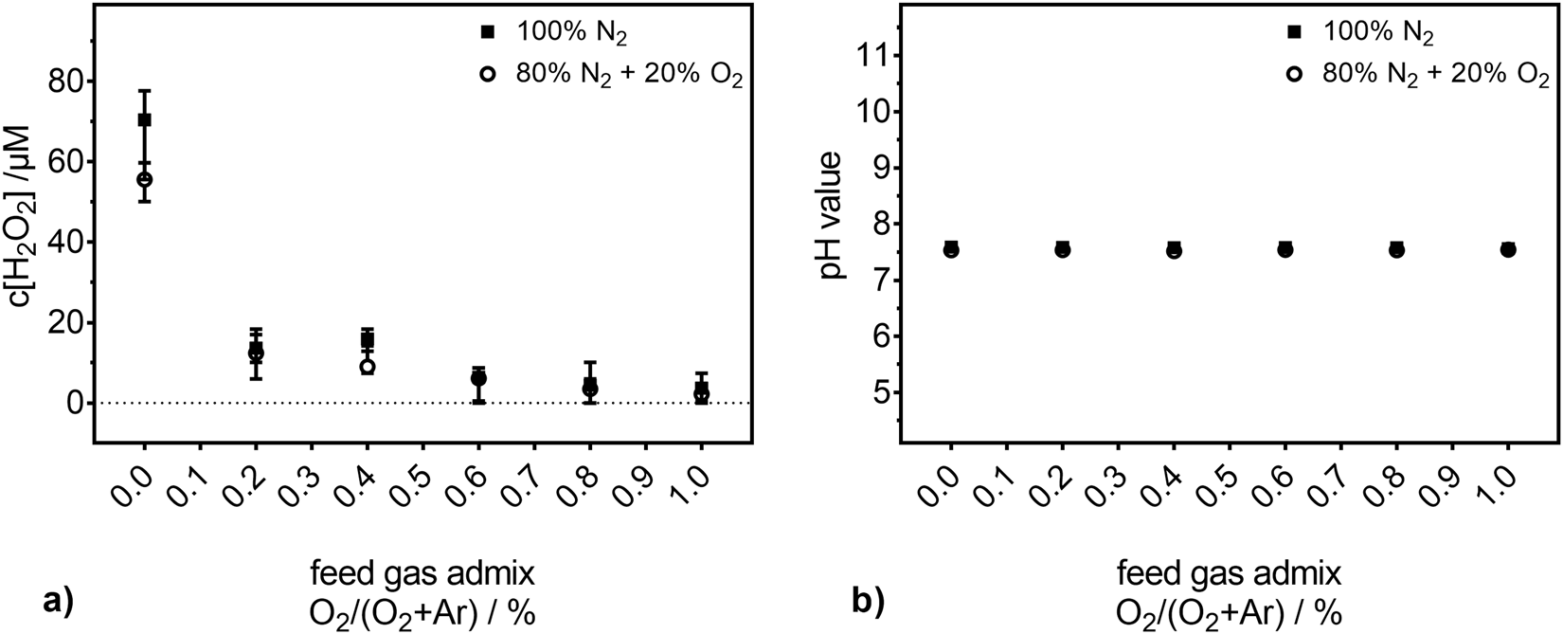
(**a**) H_2_O_2_ concentration and (**b**) stability of the pH value after direct plasma treatment (9 mm, 180 s). The values are depicted as a function of the O_2_ content in the feed gas of Ar under N_2_ or air-like atmospheres.

Even if the experiments were performed in buffered saline solutions, the pH value was monitored. In Fig. 3b, it is shown that the pH value is stable for all studied conditions, including the untreated controls (not shown).

**Figure 4:**
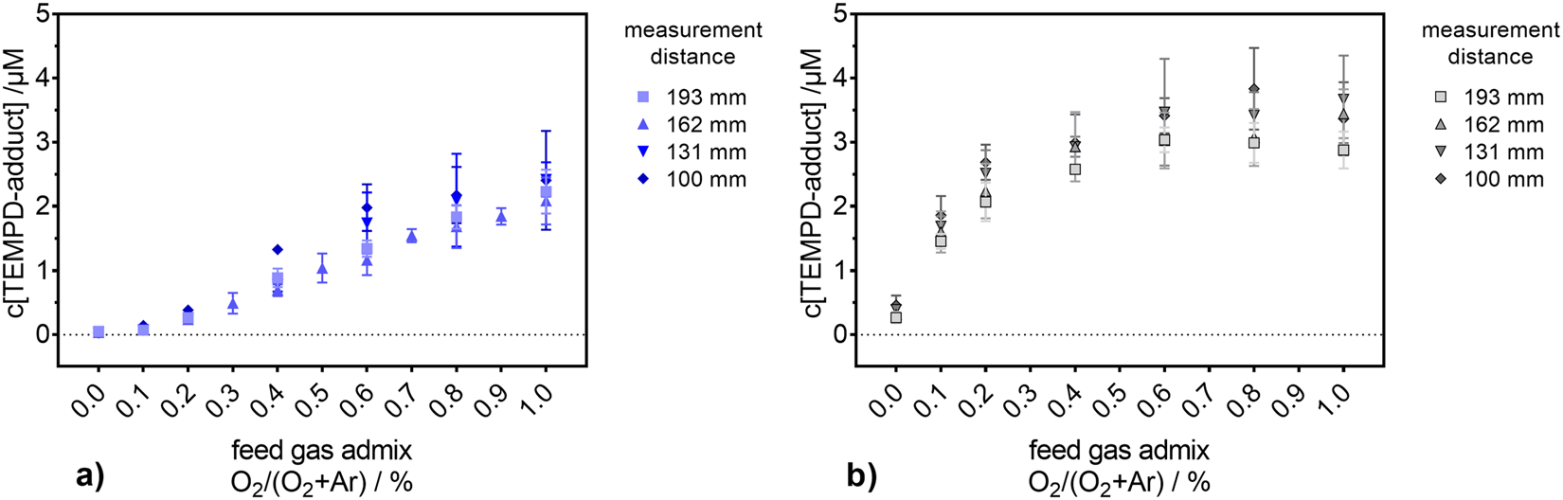
Concentration of spin probe adducts after indirect plasma treatment (d > 9 mm, 600 s). In pure N_2_ (blue, (**a**)) as well as in air-like (gray, (**b**)) environments, the plasma induces ROS in the liquid for the various distances between the plasma jet’s nozzle and the liquid studied (100, 131, 162, 193 mm).

In order to study the formation pathways of the species present in the liquid, measurements in the gas phase are necessary. O_2_(a^1^Δ_g_) is one of the possible candidates responsible for the formation of the obtained spin probe TEMPD-HCl adducts. Its density can be determined in the gas phase. Its detection by optical emission spectroscopy in the NIR may be disturbed by background radiation resulting from argon emission from the plasma. Therefore, a different experimental setup was used in order to reduce the argon emission reaching the detector (background noise). This was realized with a piece of bent glass connecting the plasma reactor and the measurement cell (see methods section for details), adversely limiting the shortest possible distance between the plasma jet’s nozzle and the measurement point. Therefore, the species density in the gas phase can only be determined at distances of at least 100 mm from the nozzle of the kinpen09. Glass connectors with different lengths were used, and the TEMPD-HCl adduct measurements in the liquid phase were, thus, repeated for the different distances between the plasma jet’s nozzle and the solutions. In Fig. 4, the obtained data is given in dependence of the O_2_ content in the feed gas for both curtain gas compositions. As the treatment of the solutions was done by the guided gas exhaust of the plasma jet that passes through the measurement cell, the resulting concentration of TEMPD-HCl adduct was found to be below the detection limit if the treatment time was kept at 180 s, as in the direct plasma treatment. Therefore, the duration of the indirect plasma treatment was increased to 600 s, in order to reach sufficient adduct concentrations. The spin probe adduct concentrations obtained after indirect plasma treatment under pure N_2_ or air-like atmosphere are given in Fig. 4a and 4b, respectively. Both conditions yield an increase of the concentration with increasing O_2_ content in the feed gas. Although different trends were found for the two surrounding atmospheres, for each curtain gas the same trend is observed for all treatment distances. In contrast to the concentrations determined after direct plasma treatments at a distance of 9 mm (see Fig. 1), here, the higher concentrations were observed for an air-like curtain gas. Furthermore, for pure N_2_ curtain gas, the concentrations of TEMPD-HCl adduct obtained after indirect plasma treatments are much lower than those gained after direct plasma treatments.

### Reactive oxygen species in the gas phase

**Figure 5:**
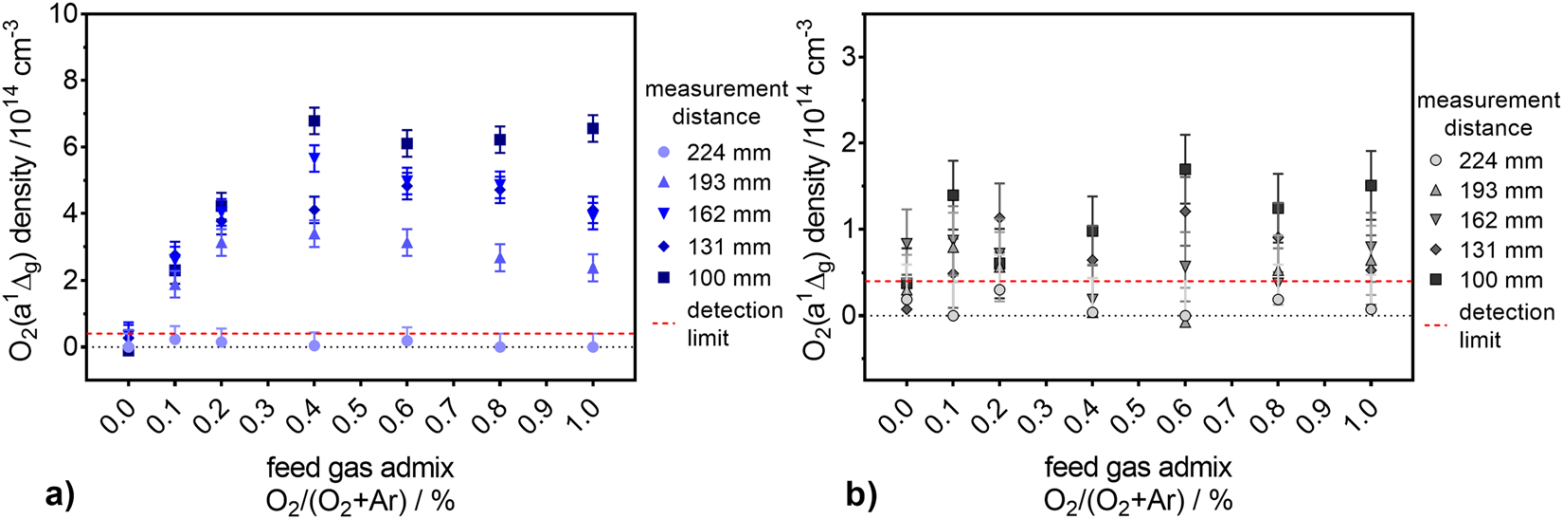
The O_2_(a^1^Δ_g_) density in the plasma jet’s exhaust for different measurement distances. The measurements were performed under pure N_2_ (**a**) and air-like environments (**b**) for several amounts of O_2_ in the feed gas. The O_2_(a^1^Δ_g_) density detection limit of 4 10^13^ cm^−3^ is indicated by red dashed lines.

To gain further insight into the species generating the spin probe adducts, gas phase measurements were performed by calibrated infrared emission (O_2_(a^1^Δ_g_)) and UV-absorption spectroscopy (O_3_). The dynamics of the O_2_(a^1^Δ_g_) density in the gas phase was studied as a function of the O_2_ content in the feed gas for different distances between the plasma jet’s nozzle and the measurement cell (see Fig. 5a,b). The O_2_(a^1^Δ_g_) density shows a clear dependence on the measurement distance; the shortest (100 mm) gap between the plasma jet’s nozzle and the point of measurement yielded the highest measured densities for both curtain gases. With increasing distance, the densities become lower, and, at the longest distance (224 mm), only small amounts of O_2_(a^1^Δ_g_) are still detectable (often below the limit of detection). Noise on low concentration points (below the limit of detection) at longer measurement distances resulted in slightly negative measurement values; these negative data points were zeroed.

In the case of N_2_ as curtain gas (see Fig. 5a), the addition of O_2_ yields a strong increase of the O_2_(a^1^Δ_g_) gas phase density (noticeable for all distances but 224 mm, where the detected densities were in the range or below the detection limit and, as so, no clear statement can be given). As expected, when working with pure Ar feed gas surrounded by pure N_2_ curtain gas, only traces of O_2_(a^1^Δ_g_), in the range or below the detection limit, were detected regardless of the distance.

For an air-like curtain gas composition (see Fig. 5b), a clear, but less pronounced dependency of the O_2_(a^1^Δ_g_) density on the distance between the plasma jet’s nozzle and the measurement cell was also observed. In great contrast to the pure N_2_ curtain condition, the O_2_ content in the feed gas when surrounded by an air-like atmosphere seems to have no effect on the O_2_(a^1^Δ_g_) gas phase density. Furthermore, the obtained O_2_(a^1^Δ_g_) densities are lower here (maximum of 1.7·10^14^ cm^−3^) compared to the N_2_ curtain gas case (maximum of 6.75·10^14^ cm^−3^).

**Figure 6:**
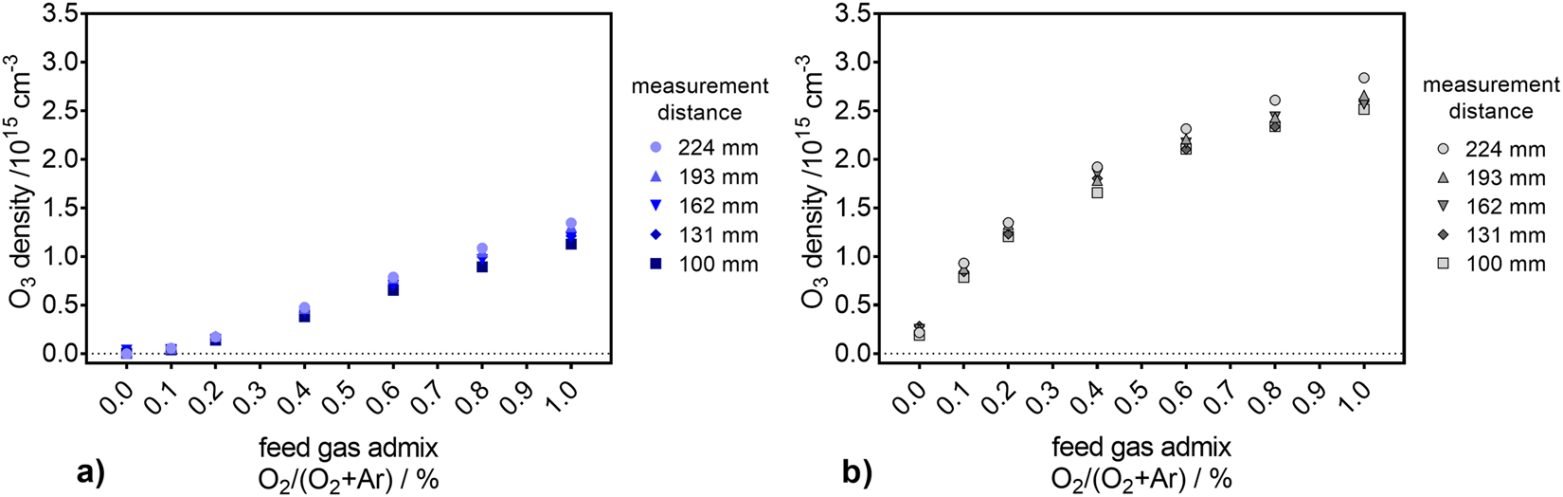
The O_3_ density in the plasma jet’s exhaust for different measurement distances. The measurements were performed under pure N_2_ (blue, (**a**)) and air-like (gray, (**b**)) environments, for several amounts of O_2_ in the feed gas.

In the gas phase measurements, O_3_ behaves differently than O_2_(a^1^Δ_g_): no distance dependency was observed for either gas curtain composition (see Fig. 6a,b). Moreover, the determined O_3_ densities are an order of magnitude higher than those measured for O_2_(a^1^Δ_g_). With increasing O_2_ content in the feed gas, the O_3_ density increases under both curtain gases, but a pure N_2_ curtain gas yields lower O_3_ densities (maximum of 1.25·10^15^ cm^−3^), independently of the O_2_ content in the feed gas. This is especially the case for the lower O_2_ admixtures, where the slope of the increase of the O_3_ density is lower when compared to the fast raise observed under the air-like curtain gas composition. Under this latter condition, the O_3_ densities reached values of up to 2.5·10^15^ cm^−3^. By comparing the maximal attained densities (obtained at 1% of O_2_ admixture in the feed gas), one can state that the N_2_ curtain gas yielded only half of the O_3_ density generated in an air-like surrounding.

## Discussion

During direct treatment of the liquid (DPBS) with the plasma plume (d = 9 mm, 180 s), a spin probe adduct of TEMPD-HCl can origin from a reaction with either O_3_, ^•^O or O_2_(a^1^Δ_g_), or a combination thereof. This adduct was measured for two different gas surroundings of the plasma plume (see Fig. 1). One of the compositions of the environment was a mixture of 80% of N_2_ and 20% of O_2_; the resulting adducts concentration increase as a function of the O_2_ content in the feed gas, saturating for O_2_ admixtures higher than 0.4%. The 100% N_2_ gas curtain results in an even stronger increase of the adducts concentration, which starts to level out at high O_2_ admixture but without reaching any saturation. It is known from literature that a pure N_2_ surrounding prevents the quenching of O_2_(a^1^Δ_g_), and, therefore, yields higher densities of O_2_(a^1^Δ_g_) in the gas phase when O_2_ is added to the feed gas^8^. In contrast, the presence of O_2_ in the surrounding environment results in an increase of the O_3_ gas density and in a decrease of the O_2_(a^1^Δ_g_) gas density^8^. Assuming a direct solvation of O_2_(a^1^Δ_g_) from the gas phase into the liquid, this predicted behaviour of the O_2_(a^1^Δ_g_) gas density could directly explain the trends observed in the liquid for the TEMPD-HCl adducts (see Fig. 1). However, other reactive species, such as ^•^OH, O_2_^•−^, or H_2_O_2_, show a different response to the modified surrounding and the varied O_2_ content in the feed gas, and ^•^OH, O_2_^•−^, and H_2_O_2_ can all contribute to the formation of at least O_2_(a^1^Δ_g_), according to reactions 1 to 3^62–65^.

1 $${O}_{2}^{\bullet -}\to {O}_{2}({a}^{1}{{\rm{\Delta }}}_{g})+\,{e}^{-}$$

2 $${O}_{2}^{\bullet -}+\,{}^{\bullet }OH\to {O}_{2}({a}^{1}{{\rm{\Delta }}}_{g})+O{H}^{-}$$

3 $${O}_{2}^{\bullet -}+{H}_{2}{O}_{2}\to {O}_{2}({a}^{1}{{\rm{\Delta }}}_{g})+O{H}^{-}+\,{}^{\bullet }OH\,$$

In contrast to the TEMPD-HCl adducts (see Fig. 1), H_2_O_2_ deposition in the liquid does not strongly respond to changes in the composition of the curtain gas (see Fig. 3a). The H_2_O_2_ concentration was only slightly higher in a pure N_2_ surrounding when no O_2_ was admixed into the feed gas. If O_2_ was present in the feed gas, the curtain gas composition did not influence the H_2_O_2_ concentration. Furthermore, H_2_O_2_, ^•^OH, and O_2_^•−^ behave similarly: with increasing O_2_ content in the feed gas their resulting concentrations decrease in a pure N_2_ surrounding. In fact, their close (chemical) relation to each other is well known^50^. ^•^OH and O_2_^•−^ themselves do not yield to a paramagnetic product with TEMPD-HCl. This, together with the different behaviour, indicates that the ^•^OH and O_2_^•−^ radicals as well as the H_2_O_2_ are not dominantly involved in the formation of O_3_, ^•^O, O_2_(a^1^Δ_g_), and, therefore, they did not contribute to the measured TEMPD-HCl adduct. Additionally, as the pH value remained constant at 7.4 during all plasma treatments (see Fig. 3b), reactions containing the hydroperoxyl (HO^•^_2_) radical can also be neglected: given its pK_a_ value of 4.8^50^, only 0.25% of the O_2_^•−^ radical is protonated. This means that O_2_^•−^ concentrations in the µM range (concentration range measured for H_2_O_2_ – see Fig. 3a) result in a low nM HO_2_^•^ concentration that can be diregarded.

Another possible mechanism of formation of O_2_(a^1^Δ_g_) in aqueous solution is through OCl^−^^11^ (see equation 4). When the treated liquid contains chloride and the plasma source is producing ^•^O in the gas phase, these two species can react to form OCl^−^^11,37^. Since the investigated solution, DPBS, contains chloride, this reaction cannot be excluded. Nevertheless, due to reaction 4, either H_2_O_2_ would be completely consumed in the solution by OCl^−^ or vice versa (depending on which species concentration is higher)^11^. The lifetime of ^•^O, as a precursor for OCl^−^, is quite short^9^ in this plasma jet, so that only low OCl^−^ concentrations are expected to be formed. Furthermore, since H_2_O_2_ is still detectable (see Fig. 3a), it is assumed that H_2_O_2_ completely consumed OCl^−^. Hence, the contribution of reaction 4 to the formation of O_2_(a^1^Δ_g_) is expected to be negligible.

4 $$OC{l}^{-}+{H}_{2}{O}_{2}\to HOCl+H{O}_{2}^{-}\to HOOCl+O{H}^{-}\to {H}_{2}O+ClO{O}^{-}\to {O}_{2}({a}^{1}{{\rm{\Delta }}}_{g})+C{l}^{-}$$

If O_2_(a^1^Δ_g_) is excluded from being responsible for the spin probe adduct formation, O_3_ is another potential candidate. O_3_ is a major fraction of the plasma produced ROS in the gas phase, when conditions allow^8,23,40,66^. O_3_ is not formed in the liquid; accordingly, it can only originate in the liquid phase through a solvation process from the gas phase.

A reaction of O_3_ and H_2_O_2_ can occur, if both are simultaneously present in the liquid. The respective set of reactions is called peroxone process (see equations 5 to 7)^67,68^. Additional UV irradiation can enhance this process. Due to the bent glass connector present in the experimental setup used for indirect plasma treatments (see Fig. 8), the amount of UV radiation reaching the liquid, and available for this specific reaction is negligible. Therefore, the UV irradiation does not have a relevant impact compared to the other ongoing processes and reactions. Under acidic conditions, the peroxone process is relatively slow, but it becomes faster for neutral or alkaline pH. Hence, in DPBS with pH 7.4, this process may take place. As the ^•^OH-adduct of BMPO and the H_2_O_2_ concentration exhibit the same behaviour for the investigated experimental conditions (see Fig. 2 and Fig. 3a), whose trend is opposite to that of the O_3_ gas density (see Fig. 6), it can be deduced that the peroxone process is not the dominant mechanism forming ^•^OH.

5 $${H}_{2}{O}_{2}\,\leftrightarrow H{O}_{2}^{-}+{H}^{+}\,$$

6 $${O}_{3}+H{O}_{2}^{-}\to H{O}_{2}^{\bullet }+{O}_{3}^{\bullet -}$$

7 $${O}_{3}^{\bullet -}+{H}_{2}O\to \,{}^{\bullet }OH+O{H}^{-}+{O}_{2}$$

Photolysis of O_3_ or H_2_O_2_ can take place in the case of a treatment at 9 mm of distance, where the plasma plume directly interacted with the liquid. During this direct plasma treatment, O_3_ can induce the formation of metastable atomic oxygen, O(^1^D), near the liquid surface (see equation 8). O(^1^D) can subsequently contribute to the formation of ^•^OH (see equation 9). The photolysis of H_2_O_2_ (see equation 10) can also result in the enhancement of the ^•^OH concentration in the liquid phase.

8 $${O}_{3}+h\nu \to {O}_{2}+O({}^{1}D)\,$$

9 $$O({}^{{\rm{1}}}D)+{H}_{2}O\to 2\,{}^{\bullet }OH$$

10 $${H}_{2}{O}_{2}+h\nu \to 2\,{}^{\bullet }OH$$

From the products of reactions 8, 9, and 10, only O(^1^D) could form the TEMPD adduct during direct plasma treatment. The peroxone process (see equations 5 to 7) as well as the photolysis of O_3_ and/or H_2_O_2_ (see equations 8 to 10) ends in the formation of ^•^OH and/or HO_2_^•^/O_2_^•−^. As these species concentration decrease with increasing O_2_ content in the feed gas whereas the TEMPD adduct concentration increases, the reactions are assumed to have minor relevance in the system.

The comparison between the behaviour as a function of the measurement distance of the density of the plasma generated species in the gas phase and of the concentration of the reactive species in the liquid phase helps to identify the species responsible for the formation of the TEMPD-HCl adduct. The curtain gas composition which yielded the highest concentration of ROS in the liquid phase was different for the different plasma treatment distances. In fact, while for the 9 mm distance treatments (direct plasma treatments), the pure N_2_ curtain yielded the highest concentration of the spin probe adducts, for longer distances (≥100 mm, indirect plasma treatment), the highest concentrations of the spin probe adducts were always obtained in the air-like surrounding. It should be noted that the concentrations of the reactive species in the liquid phase determined after direct (9 mm) and indirect (≥100 mm) plasma treatments are not directly comparable, as different treatment times were used. Actually, in order to enhance the signal-to-noise ratio in the EPR measurements, the treatment time was extended from 180 s for the 9 mm treatment gap to 600 s for the distances longer than 100 mm.

The photolysis of O_3_ or H_2_O_2_ can also take place for treatment distances longer than 100 mm, as the necessary photon energy for O_3_ and H_2_O_2_ decomposition requires wavelengths of λ < 280 nm and λ < 300 nm, respectively ^67^. Radiation below 200 nm does not reach the liquid in the investigated distances, as it is almost immediately absorbed in air. The bent shape of the indirect treatment setup also reduces considerably the incident light. Therefore, the decomposition of H_2_O_2_ and O_3_ by light absorption is assumed to be negligible in the indirect treatments (but it can be relevant in the direct treatments).

**Figure 7:**
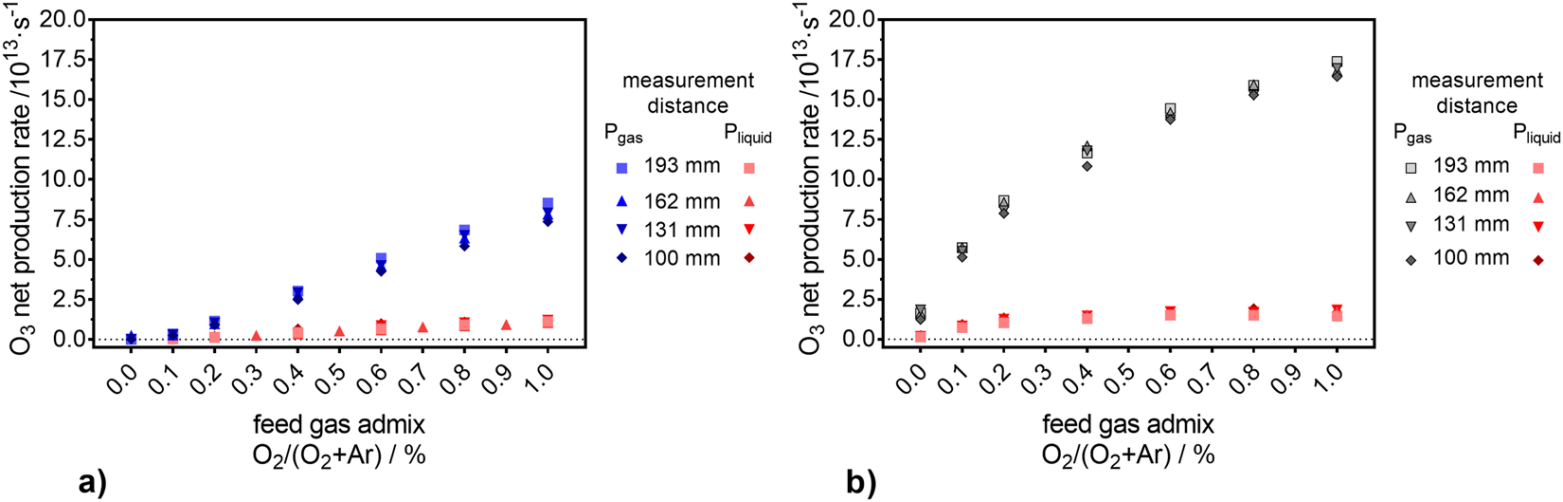
Comparison of the O_3_ net production rate in the gas and liquid phases. The net production rates in both phases are determined as a function of the O_2_ content in the feed gas under a pure N_2_ (**a**) and an air-like (**b**) environment, for different measurement distances.

For all indirect treatments, the trends of the gas phase density of O_2_(a^1^Δ_g_) are different from those observed for the concentration of TEMPD-HCl in the liquid phase (compare Fig. 4 and Fig. 5). Moreover, the O_2_(a^1^Δ_g_) gas phase density exhibits a strong dependency on the distance between the plasma jet’s nozzle and the point of measurement, while this behaviour was not observed for the TEMPD-HCl adduct formation in the liquid. Additionally, while the use of a pure N_2_ curtain gas yielded the highest O_2_(a^1^Δ_g_) gas phase densities, the highest spin probe adduct concentrations were obtained with an air-like curtain. Finally, in the latter environment, while the TEMPD-HCl adduct concentration depends on the O_2_ content in the feed gas, the gas phase O_2_(a^1^Δ_g_) density does not seem to be influenced by the O_2_ admixture into the carrier gas. Thus, it can be concluded that a solvation of gaseous O_2_(a^1^Δ_g_) cannot be responsible for the measured TEMPD-HCl adducts. ^•^O is another potential reactant with TEMPD-HCl. However, ^•^O is only present in the plasma plume and vanishes directly after leaving it, according to a model of this plasma source^9^. Therefore, for long distance indirect treatments TEMPD-HCl, adducts cannot be linked to ^•^O. Hence, the TEMPD-HCl adduct formation can be attributed to the solvation of gaseous O_3_. In fact, the O_3_ densities in the gas phase matched remarkably well the TEMPD-HCl adduct concentrations in the liquid phase, as both followed the same trends as a function of the O_2_ content in the feed gas for both gas environments, and showed higher amounts for the air-like curtain gas composition and no strict distance dependency (compare Fig. 4 and Fig. 6). For a better comparison, the net production rates of O_3_ in the gas phase and the TEMPD adduct in the liquid phase were calculated. For the gas phase, the net production rate was determined via equation (11):

11 $${{\rm{P}}}_{{\rm{g}}}({{\rm{O}}}_{{\rm{3}}})={\rm{n}}({{\rm{O}}}_{{\rm{3}}})\cdot 4.9\cdot {10}^{-4}\cdot ({{\rm{\Phi }}}_{1}+{{\rm{\Phi }}}_{2})\cdot 16.67\,{{\rm{cm}}}^{3}\cdot {{\rm{s}}}^{-1}\cdot {{\rm{slm}}}^{-1},$$

where the gas phase O_3_ density (n(O_3_)) is given in particles per cm^3^, the factor 4.9·10^−4^ is the dimensionless Henry constant of O_3_^69^, Φ_1_ and Φ_2_ are the feed gas (3 slm) and the curtain gas (5 slm) flow rates, respectively, and 16.67 is the conversion factor of standard litre per minute into the SI unit cm^−3^s^−1^. The net production rate of O_3_ in the liquid phase was calculated via equation (12):

12 $${{\rm{P}}}_{{\rm{l}}}({{\rm{O}}}_{{\rm{3}}})={\rm{c}}({{\rm{O}}}_{{\rm{3}}})\cdot {{\rm{V}}}_{{\rm{L}}}\cdot {{\rm{N}}}_{{\rm{A}}}/{{\rm{t}}}_{{\rm{treatment}}},$$

where c(O_3_) is the concentration of the TEMPD-HCl spin adduct in the liquid, V_L_ the liquid volume (5 mL), N_A_ the Avogadro constant and t_treatment_ the treatment time. Both net production rates are compared in Fig. 7a for the pure N_2_ curtain gas condition. The agreement between the gas and liquid net production rates is good, albeit a slightly increasing difference towards higher O_2_ amounts in the feed gas. This offset is due to the Henry constant of O_3_, which is relatively low – 1.2·10^−7^ M/Pa^69^. Hence, a (local) saturation of the O_3_ concentration in the liquid phase is reached relatively fast.

For an air-like surrounding, the net production rates were higher compared to the pure N_2_ gas curtain (compare Fig. 7a and Fig. 7b). This is especially the case for the O_3_ net production rate in the gas phase, where the values were more than twice as high (see Fig. 7b), and did not reach a plateau. However, the net production rate of the O_3_ deposited in the liquid showed a saturation plateau around 1.5·10^17^ s^−1^. Hence, for an air-like atmosphere, the trends of both net production rates curves did not agree completely; again, this is caused by the limited solvation of O_3_ in aqueous solutions.

It can be concluded that the similarity of the net production rates in both phases allows the identification of the oxygen species responsible for the formation of the TEMPD-HCl adducts in the liquid upon indirect plasma treatments. In fact, for treatment distanceslonger than 100 mm, the spin probe adduct is formed by reaction with O_3_. However, for direct plasma treatments, when the plasma plume interacts directly with the liquid, such statement cannot be given, since the detection of at least one of the potential reactants with TEMPD-HCl, namely O_2_(a^1^Δ_g_), is not experimentally feasible at this distance.

13 $${O}_{3}+O{H}^{-}\to H{O}_{2}^{\bullet }+{O}_{2}^{\bullet -}\,$$

14 $$H{O}_{2}^{\bullet }\leftrightarrow {H}^{+}+{O}_{2}^{\bullet -}\,p{K}_{a}=4.8$$

15 $${O}_{3}+O{H}^{-}\to H{O}_{2}^{-}+{O}_{2}$$

16 $${H}_{2}{O}_{2}^{\,}\leftrightarrow {H}^{+}+H{O}_{2}^{-}\,p{K}_{a}=11.6\,$$

17 $${O}_{3}+O{H}^{-}\to {O}_{3}^{\bullet -}+\,{}^{\bullet }OH$$

18 $${O}_{3}+{O}_{2}^{\bullet -}\to {O}_{3}^{\bullet -}+{O}_{2}$$

19 $${O}_{3}^{\bullet -}+{H}_{2}O\to {}^{\bullet }OH+O{H}^{-}+{O}_{2}$$

20 $${O}_{3}+C{l}^{-}\leftrightarrow {O}_{3}^{-}+Cl\,\approx 70 \% $$

21 $${O}_{3}+C{l}^{-}\to OC{l}^{-}+{O}_{2}\,\approx 30 \% $$

The O_3_ decomposes via ions, hydroxide (OH^−^)^70^, chloride (Cl^−^)^71^, or O_2_^•−^ ^70^ in aqueous or saline solutions^71^ (see reactions 13 to 21). For instance, ^•^OH (see equations 17 and 19) or even OCl^−^ can be formed by interaction of O_3_ with chlorite ions (see equations 20 and 21). However, the formation of OCl^−^ due to reaction 21 is subordinated, as only 30% of the formed product yields OCl^−^ ^71^. It should be noted, though, that, in other publications^72,73^, especially in Liu *et al*.^74^, this pathway is mentioned as the main formation pathway of OCl^−^. Hence, dissolved O_3_, as a gaseous precursor of other oxidizing molecules in a plasma treated liquid, is of great relevance for plasma liquid chemistry in the context of biomedical applications of plasmas, especially for jet-like plasmas where a strong stirring of the liquid occurs.

## Conclusion

The present study has yielded relevant insight for the medical application of plasma treatment. By varying the treatment distance, the composition, and, therefore, the oxidizing potential of the cocktail of generated species can be modulated. It was shown that during a direct plasma treatment, the role of ^•^O cannot be excluded, but in the case of longer treatment distances, or treatments with just the plasma gas exhaust, O_3_ is the main active component. O_2_(a^1^Δ_g_) is also formed during plasma treatment. The O_2_(a^1^Δ_g_) gas density is an order of magnitude lower than the O_3_ gas density. Moreover, according to their respective Henry constants, also the solubility of O_2_(a^1^Δ_g_) is slightly lower. Thus, it was elucidated that, for the investigated experimental conditions, O_3_ is the dominating species, being originated in the gas phase and subsequently transported to the liquid phase by solvation. By correlating the behaviour of different ROS, several reaction pathways were excluded or their importance for the proceeding reactions was weighted.

## Methods

### Plasma source

**Figure 8:**
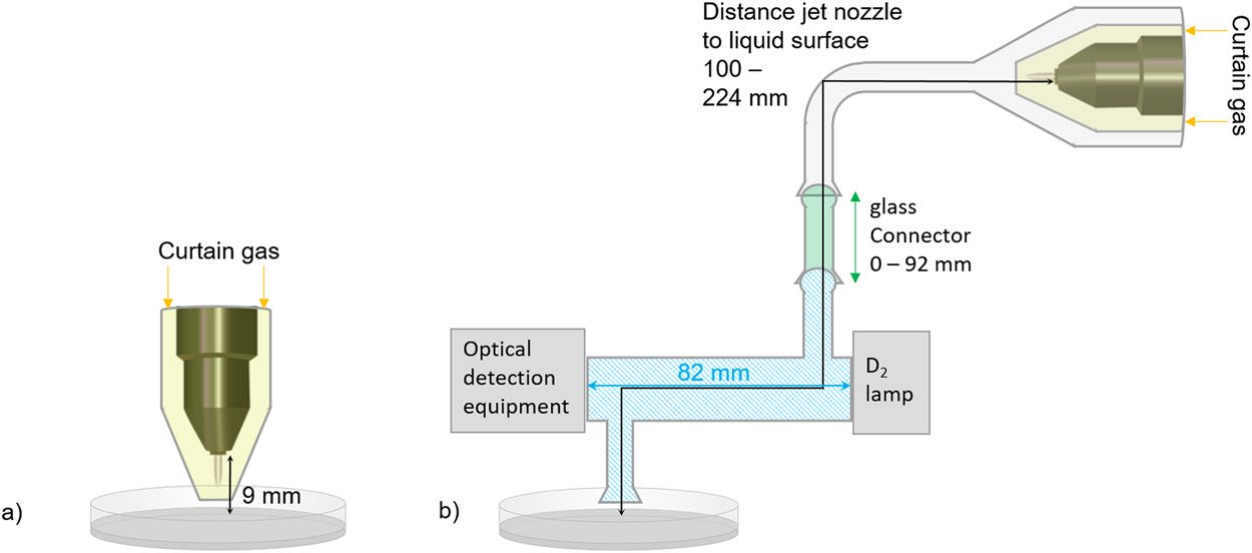
Experimental setup of the plasma treatment of liquid samples under controlled environment. Direct plasma treatment (**a**) was performed for a 9 mm distance between the plasma jet’s nozzle and the liquid surface. In (**b**), the modified setup for the investigation of O_2_(a^1^Δ_g_) and O_3_ is shown. To avoid direct or scattered light from the plasma interfering with the measurement of O_2_(a^1^Δ_g_) and O_3_, the kinpen09 with its curtain gas device (yellow-shaded area, gas inlet indicated by the yellow arrows) was enclosed in a glass capsule connected to the measurement cell through a bent glass tube. Glass connectors (green-shaded area) with different lengths (up to 92 mm, as indicated by the green double-headed arrow) were added in-between the measurement cell and the bent glass tube. The optical path length of the measurement cell (blue-shaded area) was 82 mm, as indicated by the blue double-headed arrow. The optical detection equipment used for measuring O_2_(a^1^Δ_g_) and O_3_ (InGaAs detector + filter and UV/VIS spectrometer + filter + convex lens, respectively) was placed at one end of the measurement cell. For the indirect plasma treatment (**b**), the distance between the plasma jet’s nozzle and the liquid surface varied in the range 100–224 mm (as indicated by the black double-headed arrow).

In this study, an argon atmospheric pressure plasma jet was investigated. This plasma source was a commercial plasma jet called kinpen09 (neoplas GmbH, Greifswald, Germany). It consists of a ceramic capillary (1.6 mm in diameter) with a centred rod electrode inside, and a grounded ring electrode close to the end of the capillary. The rod electrode was driven at a frequency of around 1 MHz. The dissipated power in the plasma was of 1.1 W^75^. The working gas flow was set to 3 standard litres per minute (slm); the carrier gas was argon with up to 1% of molecular oxygen admixture (argon N50, oxygen N48, Air Liquide, Paris, France). A controlled environment during the plasma treatment was provided by the application of a curtain gas device (see yellow-shaded areas in Fig. 8) fed with molecular nitrogen or synthetic air as gases (gas inlet indicated by yellow arrows), at a total gas flow rate of 5 slm (oxygen N48, nitrogen N50, Air Liquide, Paris, France). A detailed study of the effect of the curtain gas on the plasma jet can be found in previous publications^58,60^.

The liquid treatment took place in Petri dishes of 60 mm in diameter filled with a liquid volume of 5 mL of Dulbecco’s phosphate buffered saline solution (DPBS). The direct treatment was performed for a distance of 9 mm between the liquid surface and the end of the capillary, which is also called the plasma jet’s nozzle (see Fig. 8a), for 180 s.

### Gas phase diagnostics

For the gas phase diagnostics, a modified setup had to be used. This setup is schematically shown in Fig. 8b). To avoid scattered light or direct light from the plasma interfering with the measurements, the plasma jet cannot be connected directly to the measurement cell. As so, the plasma jet, including the curtain gas device, was enclosed in a glass capsule connected to the measurement cell through a bent glass tube. The measurement cell has an outlet for the exhaust, which was used to treat the liquid.

In order to study the role of O_2_(a^1^Δ_g_) and O_3_, the distance between the plasma jet’s nozzle and the measurement cell was varied by adding, in-between the measurement cell (blue-shaded area in Fig. 8b) and the bent glass tube, glass connectors (green-shaded area in Fig. 8b) with different lengths (up to 92 mm, as indicated by the green double-headed arrow in Fig. 8). The investigated distances between the end of the capillary of the plasma jet and the point of measurement of O_2_(a^1^Δ_g_) and O_3_ were 100 mm, 131 mm, 162 mm, 193 mm, and 224 mm (as indicated by the black double-headed arrow in Fig. 8).

The O_2_(a^1^Δ_g_) density was measured via optical emission spectroscopy in the near infrared (NIR) region at 1270 nm, by using an InGaAs detector (Judson model J22D-M204) and a 19 nm band pass interference filter (Andover 200FC39-25/1270)^54^. The signal collected by the detector was amplified and monitored with an oscilloscope (Tektronix DPO 2024B). The detector was placed behind a calibrated detection cell (blue-shaded area in Fig. 8b) equipped with quartz windows. This cell has a path length of 82 mm (indicated by the blue double-headed arrow in Fig. 8b)^76^. The used system has a O_2_(a^1^Δ_g_) density detection limit of 4 10^13^ cm^−3^.

The O_3_ density was measured by ultraviolet absorption spectroscopy at 254 nm. A deuterium lamp with a broadband spectrum between 180 and 400 nm was used as UV source (OceanOptics, DH-2000-BAL). The UV radiation was guided through the same measurement cell that was used for the O_2_(a^1^Δ_g_) density measurements. For the O_3_ absorption measurements, this cell was used as an absorption cell (path length of 82 mm) and the intensity of the transmitted radiation was measured by an UV/VIS spectrometer (avantes) with a filter (254 nm) and a convex lens (f = 35) placed in front of it. For deduction of the O_3_ density, the ratio of the intensity (I) transmitted when the plasma was ignited and the intensity (I_0_) transmitted when the plasma was not ignited was calculated. The O_3_ density was calculated from the Beer’s law (equation 22) with the cross section σ = 1.14·10^−17^ cm² ^77^ and the path length L = 82 mm. The O_3_ concentration is assumed to be constant across the absorption path length.

22 $$n({O}_{3})=\frac{\mathrm{ln}(\,{I}_{0}/I)}{\sigma \,\cdot L}$$

### Liquid phase analytic

For the liquid analysis, the pH values were measured electrochemically via a pH meter (SevenMulti™ S47, Mettler-Toledo International Inc., Columbus, OH, USA) equipped with a pH electrode (InLab® Micro, Mettler-Toledo International Inc., Columbus, OH, USA).

The hydrogen peroxide (H_2_O_2_) concentration was determined by commercially available test stripes (Merckoquant 110011, Merck) enhanced with a camera read out (digital microscope camera with 1.3 million pixels, zoom factor 10 to 200x, Conrad, Germany). The camera was in a dark box with defined constant light conditions in order to precisely analyse the colour of the sensor part of test stripes by determination of the red-, green- and blue-values^17^. Prior to each experiment, a calibration was conducted, where the related colour values of hydrogen peroxide concentrations in the range of 0–180 µM (0–6 mg/L) were determined.

The electron paramagnetic resonance (EPR) spectroscope used in this work is an X-band (equals to microwave frequency of 9.87 GHz) EPR (EMXmicro, Bruker BioSpin GmbH, Rheinstetten, Germany) with the resonator ER 4119HS (Bruker BioSpin GmbH, Rheinstetten, Germany). The applied instrument’s parameters of the EPR spectroscope were the same for all measurements: modulation frequency of 100 kHz, modulation amplitude of 0.1 mT, microwave power of 5.024 mW, receiver gain of 30 dB, time constant of 0.01 ms. The magnetic field scan was adjusted according to the sample measured; in this work, a range of 10 mT was used.

The measurement procedure followed always the same protocol, including performing measurements in triplicates for each sample. Prior to a measurement, the spin trap solution was prepared and 50 µL of the untreated solution were taken as control. All samples, treated and untreated, were pipetted into a borosilicate glass tube (125 mm length, 0.8525 mm inner diameter) for the measurement. From the plasma treatment to the measurement, a few minutes were needed due to handling reasons. This delay time was always fixed to four minutes. The spectrum of the untreated control was subtracted from the measured EPR signal. The evaluation of the EPR spectra was performed by assistance of the evaluation software (Xenon software with Xenon Spin Counting module, Bruker BioSpin, Rheinstetten, Germany). As the spectroscope was calibrated, absolute spin numbers could be gained.

In the presented work, 5-tert-Butoxycarbonyl-5-methyl-1-pyrroline-N-oxide (BMPO, 2 mM) was used as a spin trap for ^•^OH and O_2_^•−^ radicals, and 2,2,6,6-tetramethyl-4- piperidone hydrochloride (TEMPD-HCl, 100 mM) was used as a spin probe for ^•^O, O_2_(a^1^Δ_g_), or O_3_. The used BMPO concentration was chosen based on previous studies^19^ using the exactly same plasma source, where higher concentrations yielded similar adduct concentrations in the µM range. Therefore, the chosen concentration of 2 mM is high enough to trap all detectable radicals. The TEMPD-HCl concentration used in the study was based on literature^78^; furthermore, the used concentrations were far higher than the detected adduct concentration, so that the spin trap was offered in excess.

## Data Availability

The datasets generated during and/or analysed during the current study are available from the corresponding author on reasonable request.
